# Reduced resilience of brain gray matter networks in idiopathic generalized epilepsy: A graph-theoretical analysis

**DOI:** 10.1371/journal.pone.0212494

**Published:** 2019-02-15

**Authors:** Daichi Sone, Masako Watanabe, Norihide Maikusa, Noriko Sato, Yukio Kimura, Mikako Enokizono, Mitsutoshi Okazaki, Hiroshi Matsuda

**Affiliations:** 1 Department of Psychiatry, National Center of Neurology and Psychiatry, Tokyo, Japan; 2 Integrative Brain Imaging Center, National Center of Neurology and Psychiatry, Tokyo, Japan; 3 Department of Radiology, National Center of Neurology and Psychiatry, Tokyo, Japan; McGill University, CANADA

## Abstract

**Purpose:**

The pathophysiology of idiopathic generalized epilepsy (IGE) is still unclear, but graph theory may help to understand it. Here, we examined the graph-theoretical findings of the gray matter network in IGE using anatomical covariance methods.

**Materials and methods:**

We recruited 33 patients with IGE and 35 age- and sex-matched healthy controls. Gray matter images were obtained by 3.0-T 3D T1-weighted MRI and were normalized using the voxel-based morphometry tools of Statistical Parametric Mapping 12. The normalized images were subjected to graph-theoretical group comparison using the Graph Analysis Toolbox with two different parcellation schemes. Initially, we used the Automated Anatomical Labeling template, whereas the Hammers Adult atlas was used for the second analysis.

**Results:**

The resilience analyses revealed significantly reduced resilience of the IGE gray matter networks to both random failure and targeted attack. No significant between-group differences were found in global network measures, including the clustering coefficient and characteristic path length. The IGE group showed several changes in regional clustering, including an increase mainly in wide areas of the bilateral frontal lobes. The second analysis with another region of interest (ROI) parcellation generated the same results in resilience and global network measures, but the regional clustering results differed between the two parcellation schemes.

**Conclusion:**

These results may reflect the potentially weak network organization in IGE. Our findings contribute to the accumulation of knowledge on IGE.

## Introduction

Idiopathic generalized epilepsy (IGE) is a common subgroup of epilepsy that encompasses several well-established epilepsy syndromes with common features such as primarily generalized seizures and epileptic discharges on electroencephalography (EEG) [1, 2]. Although patients typically show normal findings on conventional MRI, statistical and advanced methods have revealed various relevant abnormalities in IGE [3]. Neuropsychological studies suggest frontal dysfunction, whereas advanced neuroimaging mostly report the involvement of the thalamus and frontal cortex [3]. While a multicenter worldwide study reported thalamic volume reduction and reduced cortical thickness in bilateral precentral gyri in IGE [4], a recent meta-analysis revealed volumetric alterations in the thalami, basal ganglia, frontal lobes, corpus callosum, hippocampus, insula, and overall brain volumes [5]. However, the pathophysiology of IGE remains to be elucidated.

Graph theory was originally a mathematical method to analyze various networks that are regarded as consisting of nodes and edges [6]. In the neurosciences, including the study of epilepsy, multiple neuroimaging methods can be used for graph-theoretical analyses by modeling the brain as complex networks consisting of nodes and edges [6, 7]. For example, diffusion tensor imaging (DTI) provides graph metrics using white matter tractography, whereas functional MRI assesses the connectivity of blood oxygenation level-dependent signals [6]. Although these methods can be used to help identify biomarkers that are potentially clinically useful, the interpretation of the results is often complex due to the partially heterogeneous findings from different methodologies [8]. Therefore, further studies using new and different modalities may help to expand our understanding of this field.

Anatomical covariance analysis is one of the important and useful methods that draws on graph theory [9], and a growing number of studies have used this approach to obtain findings pertinent to epilepsy [10–13]. This method can extract measures of inter-regional connectivity from the covariance patterns of brain morphology and may provide more knowledge on neuropsychiatric diseases when used in combination with functional or diffusion imaging [9, 14]. In particular, evaluation of neural networks in the brain would be useful for epilepsy, given its status as a network-level disorder [7]. The basis of anatomical covariance analyses is the inter-regional correlations of gray matter volumes or thicknesses, which are considered to represent brain regional connectivity.

For IGE specifically, one previous graph-theoretical study revealed reduced resilience of the cerebral blood flow (CBF) networks and thalamic hypoperfusion [15]. Additionally, connectivity between cortical thickness and thalamic volumes was also investigated [16]. However, compared with the evidence accumulated for temporal lobe epilepsy (TLE) [12, 13], little is known about whole-brain gray matter covariance networks in adults with IGE. Given that different graph-theoretical methods have yielded both concordant and conflicting results in epilepsy [17], it would be meaningful to reveal the characteristics of gray matter connectivity in IGE.

In this study, we applied graph-theoretical techniques to the whole gray matter network in IGE and compared the results with those of previous studies.

## Materials and methods

### Subjects

We recruited 33 patients with IGE (25 women, 8 men; mean ± SD age, 26.4 ± 7.4 years) at our institute between March and August 2016. IGE was diagnosed if the following criteria were met: (1) the presence of primarily generalized seizures with no focal symptoms, (2) diffuse (poly)spike-wave complex on interictal conventional scalp EEG, and (3) no focal abnormality on conventional MRI. Of the 33 patients, 16 had generalized tonic-clonic seizures alone, 12 had myoclonic seizures, and 5 had typical absence seizures in addition to generalized tonic-clonic seizures. The mean ± SD seizure onset age was 15.6 ± 4.8 years.

We also recruited 35 age- and sex-matched healthy volunteers as controls (26 women, 9 men; mean ± SD age, 27.2 ± 6.3 years). Between the IGE and control groups, there were no significant differences in age (p = 0.64 by unpaired t-test) or sex (p = 0.89 by Pearson's χ^2^).

We obtained written informed consent from all participants, and the study was approved by the institutional review board at the National Center of Neurology and Psychiatry Hospital (Tokyo, Japan).

### MRI acquisition

MRI scans for all participants were performed using a 3.0-T MR system with a 32-channel coil (Achieva, Philips Medical Systems, Best, The Netherlands). We obtained three-dimensional sagittal T1-weighted MP-RAGE (magnetization-prepared rapid acquisition with gradient echo) images with the following protocol: repetition time/echo time, 7.2/3.4 ms; field of view, 26 × 26 cm; matrix, 384 × 384; effective slice thickness, 0.6 mm with no gap; number of slices, 300; and number of excitations, 1.

### MRI processing

The imaging data were analyzed in Statistical Parametric Mapping 12 software (SPM12; http://www.fil.ion.ucl.ac.uk/spm/) running in MATLAB 2014a (The Mathworks, Natick, MA, USA). The T1-weighted images were segmented into gray matter, white matter, cerebrospinal fluid, and other non-brain tissues by a unified tissue segmentation procedure after correction for nonuniformity of image intensity. These segmented gray and white matter images were then spatially normalized to a customized template in the standardized anatomic space (i.e., MNI space) by using the DARTEL (diffeomorphic anatomical registration using the exponentiated Lie algebra) toolbox [18]. Each image was then modulated by the Jacobian determinants derived from the spatial normalization by DARTEL and spatially smoothed with a 6-mm full-width at half-maximum Gaussian kernel to decrease spatial noise and compensate for the inexactitude of normalization. Although controversy persists about the appropriate size of smoothing and its effect on statistics, we chose the value of 6 mm, considering the advances in the DARTEL registration algorithm [19].

### Graph-theoretical analysis

**Fig 1:**
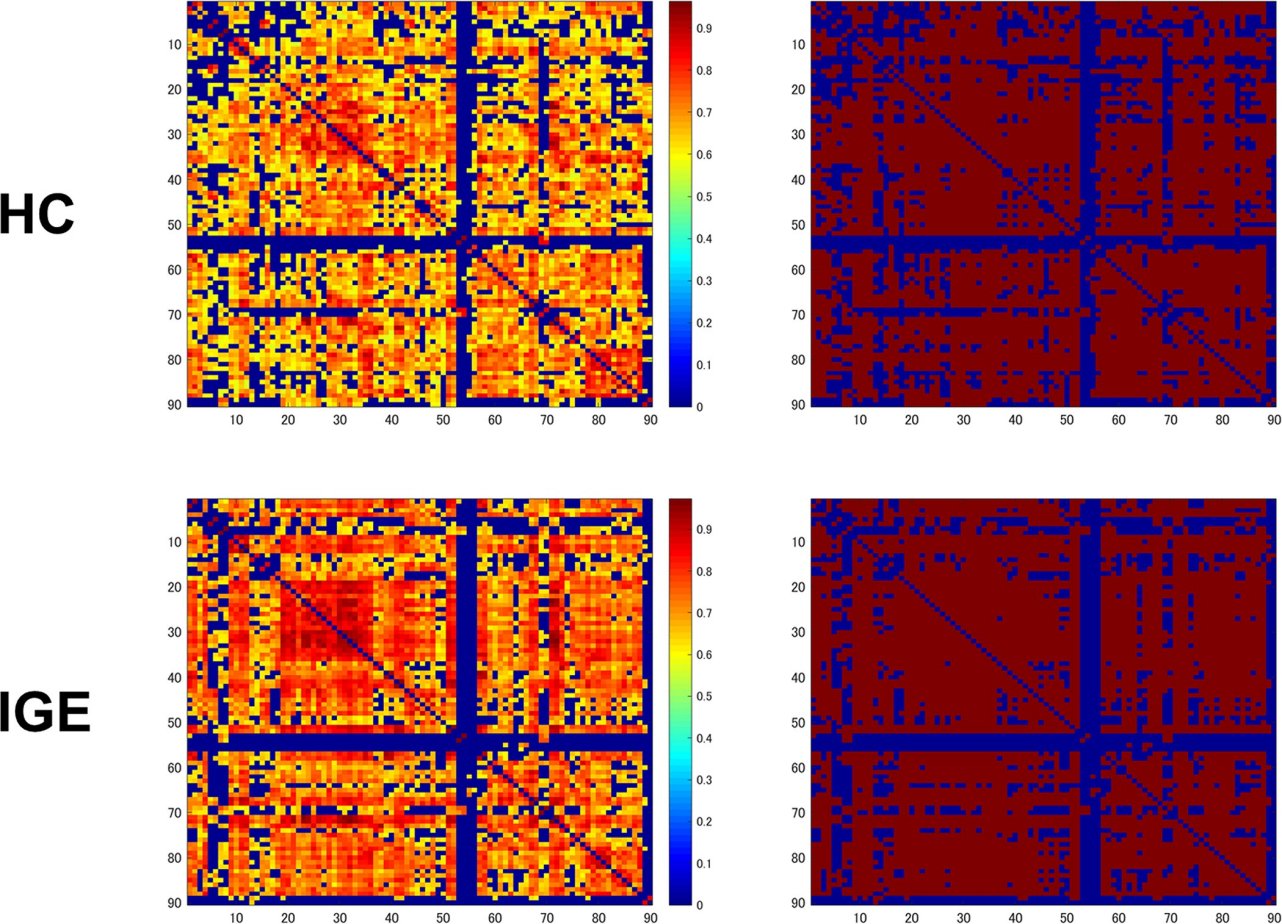
**The correlation matrices of the control and IGE groups (left, thresholded; right, binarized).** The X/Y axes denote the 90 ROIs from the AAL template.

Graph Analysis Toolbox (GAT) was used for the graph-theoretical analysis in this study [20]. GAT is open-source software that provides a graphical user interface to facilitate analyses and comparisons of anatomical brain networks. We used the normalized gray matter images of both groups in GAT running within MATLAB 2014a. The available region-of-interest (ROI) parcellation scheme for GAT comprised the 90 cortical and subcortical regions from the Automated Anatomical Labeling (AAL) template [21]. In the structural covariance analysis in this article, correlational networks were constructed on the basis of gray matter volumes derived with SPM12 using an AAL whole-brain parcellation. Accordingly, GAT analyzed all 90 ROIs, and a 90 × 90 association matrix (Pearson correlation coefficient) for each group and modality was generated (Fig 1). The matrices were thresholded at multiple densities (ranging from 0.10 to 0.50 at intervals of 0.02) and converted into binary adjacency maps (Fig 1).

Subsequently, the following network metrics were calculated: clustering coefficient (*C*), a measure of the number of edges that exist between a node and its nearest neighbors; characteristic path length (*L*), the average shortest path length between all pairs of nodes as a measure of network integration; and C_rand_ and L_rand_, where C_rand_ and L_rand_ are the normalized clustering coefficient and the characteristic path length of 20 random networks, respectively. Network resilience to random failure and to targeted attack was also evaluated. Random failure was assessed by randomly removing one node from the network and measuring changes with each iteration, whereas targeted attack was assessed by removing the nodes in rank order of decreasing nodal betweenness centrality. Moreover, GAT performed nonparametric permutation tests and assessed the regional difference in clustering between the two groups.

The above analyses were fully automatic; the details of the processes have been described previously [20].

Furthermore, to enhance validity and reproducibility, we repeated the above graph-theoretical analyses using a second parcellation scheme, as with previous graph-theoretical studies [12, 22]. For the second parcellation scheme, we used 58 ROIs from the Hammers Adult atlas (http://brain-development.org/) [23, 24], excluding the ROIs of the ventricles, cerebellum, and brainstem. The ROIs that we used are contained in our dataset online.

### Statistical analyses

GAT was used to compare the areas under the curve (AUCs) of each network measure of the two groups, in which the curves extracted from thresholding across a range of densities are used. The AUC analyses were also performed for the curves of random or targeted attacks. To test the significance, either a nonparametric permutation test or a parametric t-test was performed [20]. GAT was also used to perform a one-tailed nonparametric permutation test (1,000 repetitions) to evaluate the regional differences in clustering between the two groups. The use of either a one- or two-tailed test in regional comparison is optional in the software [20], but we used a one-tailed test because of a technical issue with the software. This permutation testing adopted a shuffled assignment of each permutation group. In fact, in each repetition, the regional data of each participant were randomly reassigned to one of the two groups and thus each randomized group had the same number of subjects as the original groups [20]. To correct for multiple comparisons, a false discovery rate of p < 0.05 was deemed significant.

To assess morphological differences between the two groups, we tested the normalized gray matter images using the two-sample t-test analysis in SPM12, with age, sex, and intracranial volumes calculated by SPM12 as nuisance covariates. Additionally, we also investigated the correlations between gray matter volumes and disease duration in the IGE group, using the multiple regression model in SPM12, with age, sex, and intracranial volumes as nuisance covariates. Statistically, a height threshold of p < 0.05 (false discovery rate) was deemed significant. However, to consider the effect of subtle morphological changes on graph-theoretical results, we also reported all clusters thresholded by a height of p < 0.005 (uncorrected) in SPM12 analyses.

## Results

### Network measures

**Fig 2:**
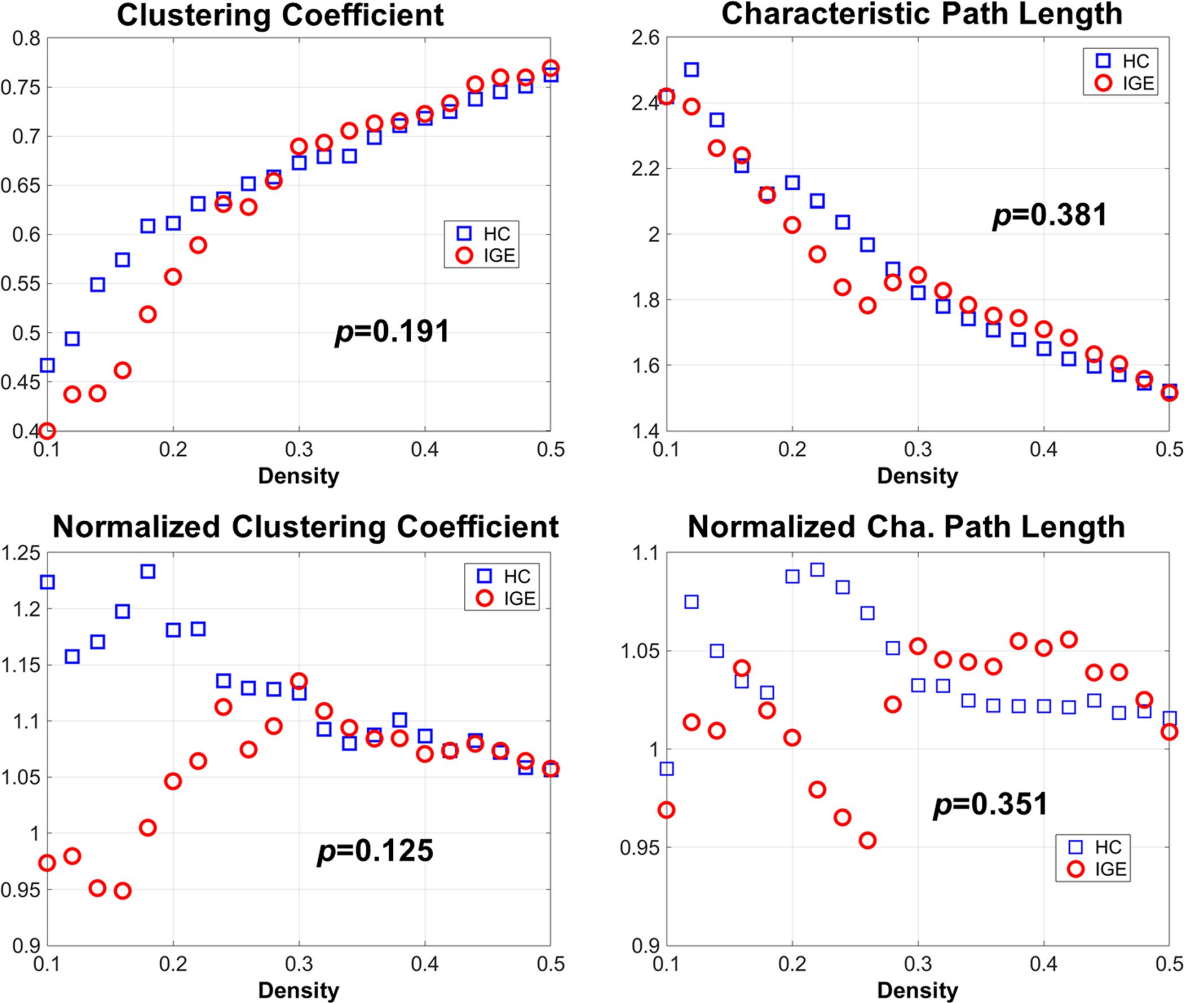
Network metrics and the p-values of AUC comparisons between the control and IGE groups. The meanings of the measures are described in the Methods section of the text.

The network measures and the comparison results are shown in Fig 2. There were no significant differences in any of the metrics, including the clustering coefficient and characteristic path length. It should be noted, however, that a lack of significant differences is not necessarily evidence of a lack of differences in the functions of whole network.

### Resilience analyses

**Fig 3:**
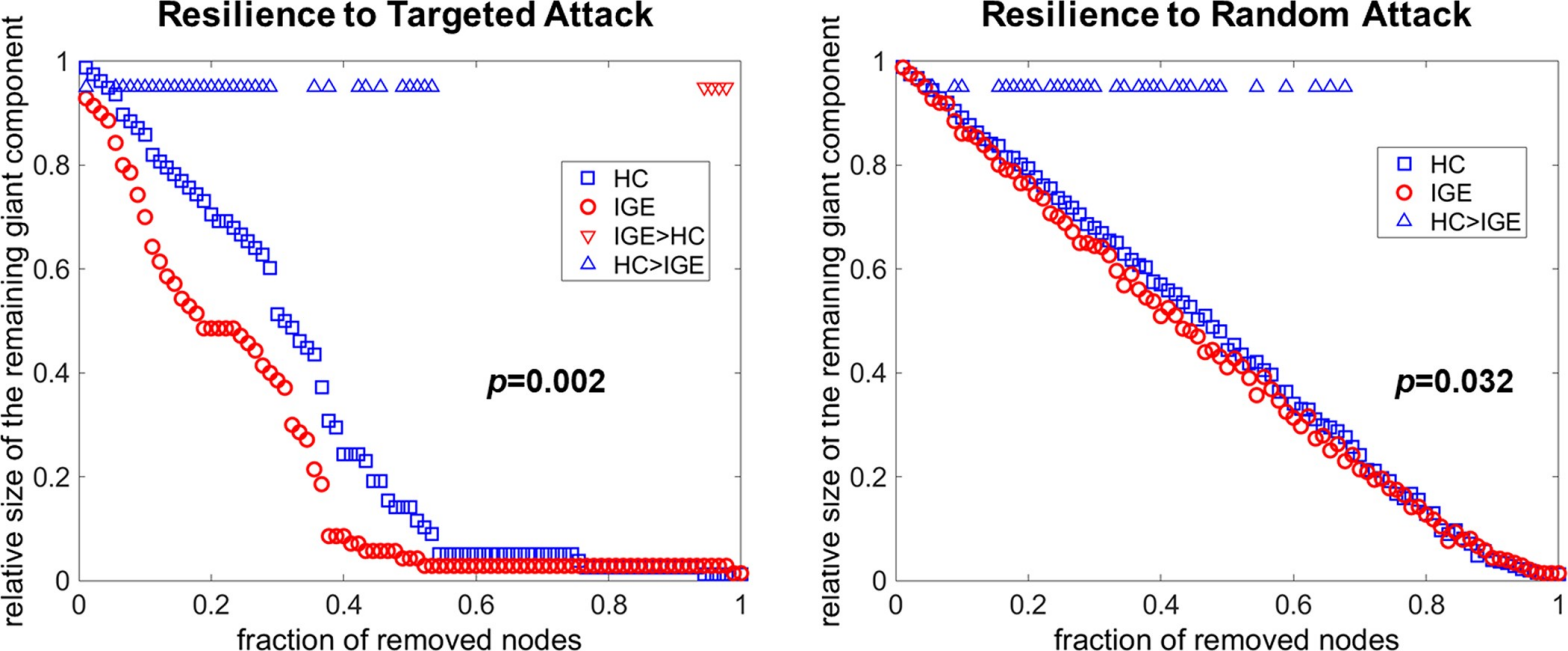
Assessments of network resilience to random failure or targeted attack. Triangles indicate significant differences between the control and IGE groups. Additionally, the displayed p-values were calculated based on AUC comparisons.

The resilience analyses revealed significantly reduced resilience of the IGE gray matter networks to both random failure and targeted attack (Fig 3). This means that an assumed removal of nodes can cause significantly more effects in the IGE gray matter networks compared with controls.

### Regional clustering

**Fig 4:**
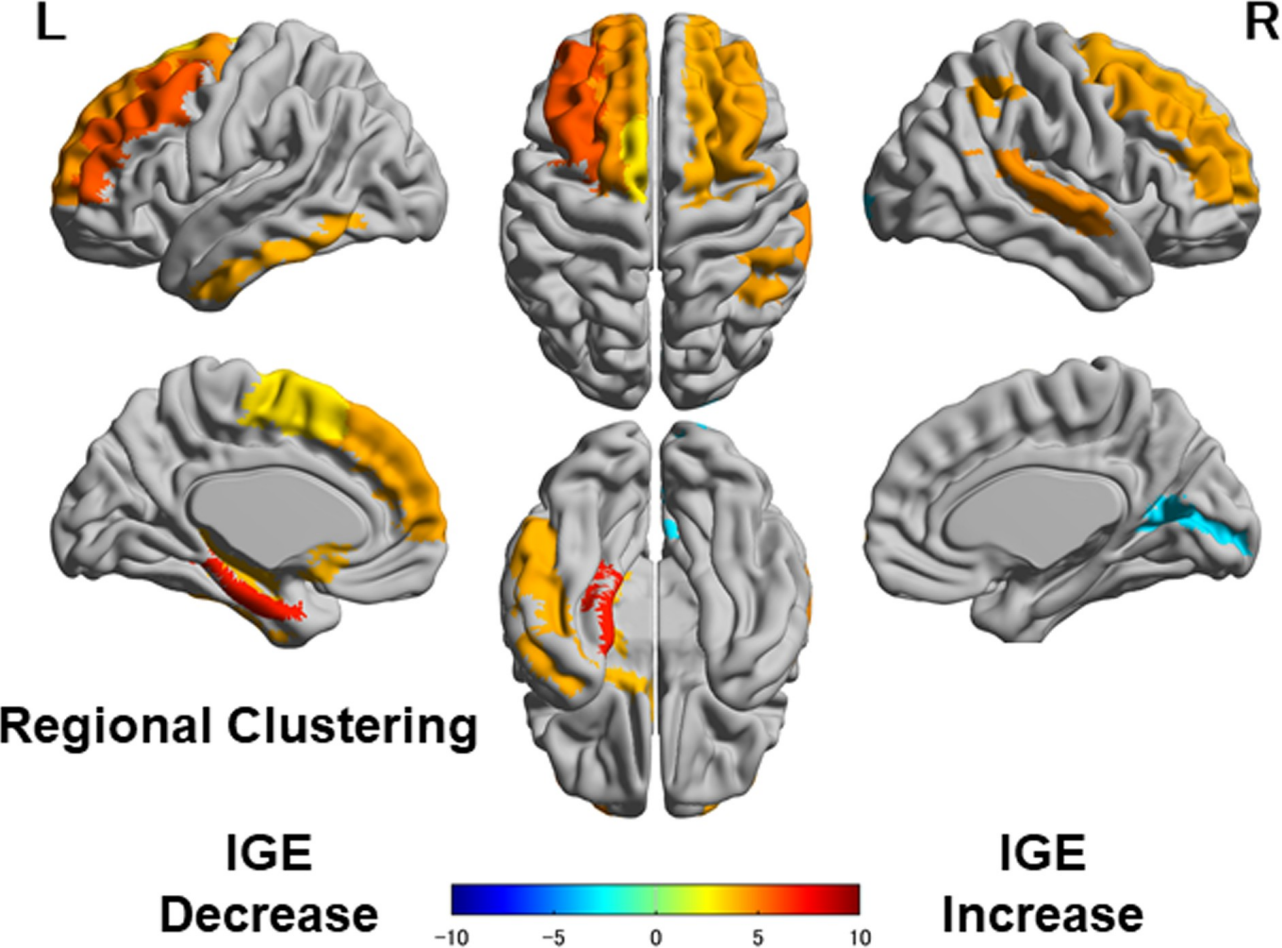
Regional comparisons of clustering between the control and IGE groups. Colored areas indicate significant differences.

As for the regional clustering, the IGE group showed an increase in the bilateral frontal lobes, temporal lobes, left hippocampus, and right marginal gyrus, and a decrease in the right occipital lobe (Fig 4). Given that the clustering coefficient represents the number of edges that exist between the nearest neighbors of each node, the current findings may be associated with altered local network densities in those areas.

### Morphological differences and correlations with disease duration

**Fig 5:**
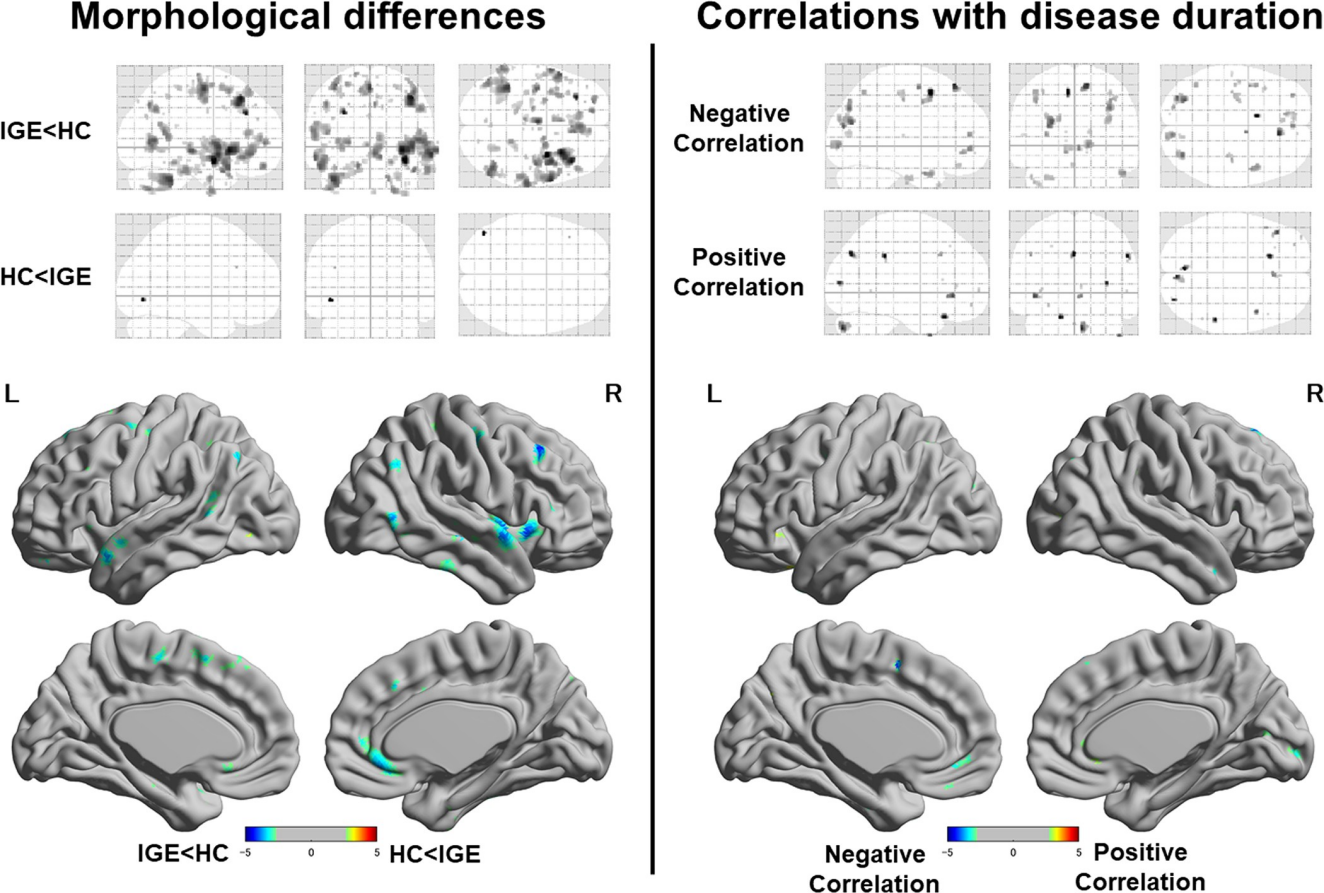
**Glass brains (upper) and T-value maps (lower) regarding the morphological differences between groups (left) and correlations with disease duration in the IGE group (right).** The reported regions are thresholded at the level of an uncorrected p < 0.005 (T > 2.65 in comparison, T > 2.76 in correlation).

At the conservative level of p < 0.05 (false discovery rate), there were no significant morphological differences between the groups and no significant correlations with disease duration. At the exploratory level of p < 0.005 (uncorrected), we found mild gray matter loss mainly in fronto-temporal lobes (Fig 5, left) and small areas, mainly in frontal and occipital lobes, of correlation with disease duration (Fig 5, right).

### Graph-theoretical analysis by the second parcellation scheme

**Fig 6:**
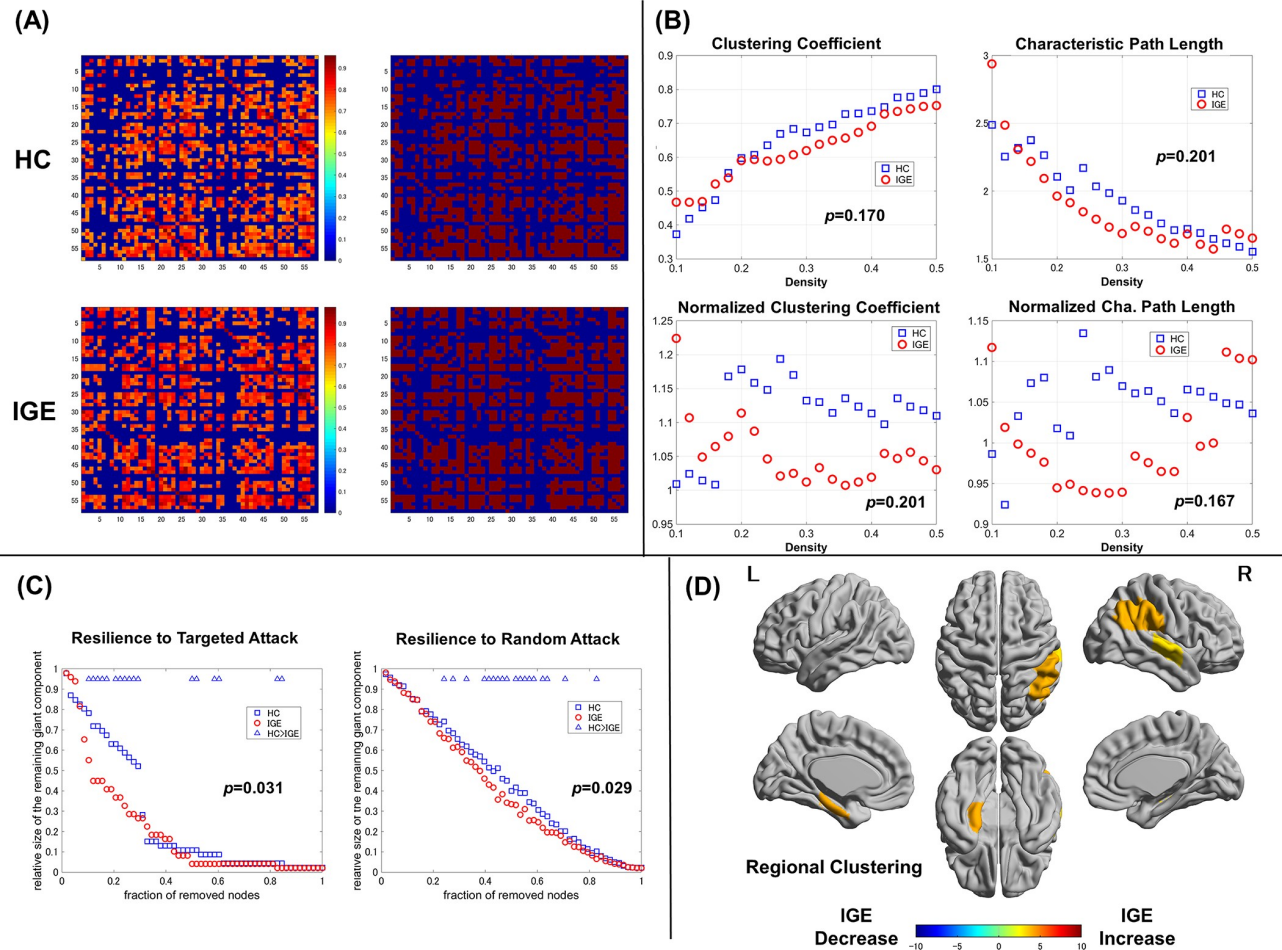
The results of the additional graph-theoretical analysis by the second parcellation scheme (58 ROIs from Hammers Adult atlas). (A) Correlation matrices, (B) network metrics, (C) resilience analyses to attacks, and (D) regional clustering changes.

Fig 6 shows the results of additional graph-theoretical analysis by 58 ROIs from Hammers Adult atlas. We found a lack of significant differences in the network metrics and significantly reduced resilience in the IGE group to both targeted and random attacks (Fig 6B and 6C), consistent with the results of the first parcellation scheme. On the other hand, the regional clustering changes were different from those of the first analysis with AAL ROIs (Fig 6D). There were no differences with regards to the frontal parcels, whereas the differences remained significant for lateral temporal and mesiotemporal clusters.

## Discussion

In this study, we used anatomical covariance methods to perform graph-theoretical analyses of gray matter structural networks in IGE. We found significantly reduced resilience to attacks, no significant changes in network measures, and several regional changes in clustering. Additionally, the reduced resilience and insignificant changes in global network metrics were reproduced by repeated analysis with another parcellation. To our knowledge, this is the first study to investigate the whole-brain gray matter network in adults with IGE. The reduced resilience is particularly meaningful because the results are in line with those of a previous study of CBF networks in IGE [15]. Reduced resilience of brain networks was also reported in TLE using gray matter [12] and CBF [25]. Basically, a human brain network is considerably more resilient to targeted attacks than a comparable scale-free network [26], and the pathologically reduced resilience could be attributed to a lack of potential alternative backup networks in the affected brain [12]. Furthermore, according to a previous functional MRI study [27], the resilience to targeted or random attacks was correlated with intelligence, and the relevant brain regions with resilience were language, memory, and emotional processing areas. Although we speculate that the reduced resilience of gray matter networks in IGE might be associated with these dysfunctions of higher-order cognition or impaired neuronal pathways by seizure activities, further investigations with cognitive examinations and individual-level analyses should be performed to clarify this issue.

Additionally, we found no significant changes in network metrics in IGE. These findings were consistent in parcellations of both the AAL and Hammers Adult atlas. The gray matter networks in IGE may have generally comparable integrations and segregations to healthy subjects, although a lack of significant differences is not necessarily evidence of a lack of differences in the functions of the whole network. Whereas many articles have reported increased path length in TLE or focal epilepsy [12, 13, 25, 28, 29], studies of IGE have found no significant changes in path length [15, 22]. Thus, our results may provide additional evidence that IGE shows no significantly changed path length in brain networks. Compared with the insignificant clustering coefficient in the current study, a previous graph-theoretical study using functional MRI data and DTI reported a decreased normalized clustering coefficient in IGE with generalized tonic-clonic seizures alone [22]. The inconsistency of clustering coefficient findings among various modalities was raised by a previous review of epilepsy [17]. The explanation for these inter-study differences could involve disease progression, effect of drugs, or sample variances [17].

Regarding regional analyses, we found several significant changes in clustering. The regional graph-theoretical metrics can represent the disease-specific brain areas. For example, a previous study of TLE revealed distinctly different patterns, in which TLE with hippocampal sclerosis showed functional segregation of the sclerotic hippocampus and MRI-negative TLE was associated with impaired connectivity of the ipsilateral temporal neocortex [30]. In our study, the increased regional clustering coefficient was found mainly in wide areas of the bilateral frontal lobes. In fact, frontal lobe dysfunction is most commonly reported in neuropsychological studies of IGE [3, 31, 32], despite the absence of obvious intellectual disability. Moreover, previous functional or diffusion neuroimaging studies of IGE also revealed abnormalities in frontal lobes [32–35]. Considering this evidence, the increased regional clustering may reflect the altered frontal connectivity with structural and functional damages in IGE. However, it is of importance that the second ROI parcellation could not reproduce the findings. The commonly altered areas were limited to the left hippocampus, a part of the right temporo-parietal lobe (increased regional clustering). Given the evidence of extra-frontal structural abnormalities early in the course of juvenile myoclonic epilepsy [36], the increased regional clustering in extra-frontal areas might be due to an altered brain developmental trajectory. Additionally, we speculate that regional metrics could be largely affected by the ROI parcellation, which defines each region of the brain in this analysis. The relationships between regional measures and ROIs remain to be elucidated in further studies.

Although the most common finding of voxel-based morphometry (VBM) in IGE is thalamic atrophy [3], we found a trend-level atrophy mainly in frontal and temporal lobes. In fact, the results of VBM studies of IGE are somewhat heterogeneous [3], with one study reporting no significant VBM difference in frontal gray matter in younger patients with juvenile myoclonic epilepsy (mean age, 24.2 years) [37]. One article reported frontal lobe atrophy as well as thalamic volume loss [38], and the anatomical connection between these structures is altered in IGE [16]. Notably, in most studies reporting thalamic atrophy, the mean ages of patients are relatively older (over 30 years) [39–41], except for two reports using teenage patients with childhood absence epilepsy and a large number of healthy controls [42, 43]. Given the very young onset of childhood absence epilepsy, we speculate that the key factor in the differing VBM results in IGE is disease duration. A few studies have reported the effect of disease duration on progressive thalamic atrophy in IGE [16, 38, 44], although we found no correlation between thalamic volume and disease duration in our cohort, possibly due to the sample size or shorter disease duration (mean age, 26.4 years; mean disease duration, less than 10 years). The interictal hypoperfusion [15, 45] and/or ictal activation [46, 47] in the thalamus may gradually cause thalamic atrophy as disease duration increases. This type of progressive atrophy has also been revealed in intractable TLE [48]. On the other hand, a later study has reported thalamic volume loss in drug-naïve initial-onset IGE patients [49]. Moreover, the above-mentioned study reporting frontal and thalamic atrophy in IGE included patients with less than 10 years of mean disease duration [38]. According to the most recent multicenter analysis with a large sample size [4], IGE shows significant thalamic volume loss and reduced cortical thickness in the bilateral precentral gyri. Although a slight volume loss in precentral gyri can also be found (Fig 5), our cohort appears to have different morphological features from the large-scale analysis. In particular, the lack of evident thalamic atrophy should be considered for careful interpretation and generalization of the graph-theoretical results.

This study has several limitations. Binary thresholding is a common method in this field but could be a source of bias [50]. According to the literature [50], the range of thresholds to sum across should be chosen with care. The inclusion of low thresholds is likely to include effects from false positives, whereas the inclusion of very high thresholds will include measurements of highly disconnected networks that are not reflective of the true connectome. In addition, there can be a failure to detect true group effects if the effect only manifests in a limited range of thresholds. We selected the range of 0.10 to 0.50 at intervals of 0.02 as thresholds. As for the maximum threshold, high thresholds, particularly those exceeding a density of 0.50 [51], have been found to be problematic because of the sparse and highly disconnected networks they yield. Our minimum threshold (i.e., 0.10) is also controversial, although many studies of anatomical covariance neuroimaging use a density of around 0.10 as the minimum [12, 13, 51, 52]. The selection of thresholding ranges is an important limitation of this study, but we consider our thresholding range to be appropriate compared with other similar articles and to not be far off the mark.

Furthermore, the results should be interpreted with caution in regard to the use of one-tailed permutation test, which might have reduced statistical significances. However, we confirmed most of the results by two different parcellation schemes, and several previous studies also adopted one-tailed test in GAT [10, 25, 53].

In addition, IGE consists of several different epilepsy syndromes, and our IGE cohort combined patients with different seizure types into one group. However, the term “IGE” is still accepted [1], and some common features and similar underlying mechanisms can be supposed. Moreover, we should consider the effects of antiepileptic drugs and the relatively small sample size when interpreting the present results.

## Conclusion

We have identified several features of gray matter networks in the IGE brain, including reproducible reduced resilience to attacks and no significant metric changes in the clustering coefficient or characteristic path length. Increased regional clustering was found mainly and widely in frontal lobes, but it was not confirmed by the second analysis. In particular, these results would reflect the potentially weak network organization of IGE. Our findings contribute to the accumulation of knowledge on IGE.
